# Trends in abdominal aortic aneurysm-related mortality in Brazil, 2000-2016: a multiple-cause-of-death study

**DOI:** 10.6061/clinics/2021/e2388

**Published:** 2021-01-11

**Authors:** Augusto Hasiak Santo, Pedro Puech-Leão, Mariana Krutman

**Affiliations:** I Associate Professor (retired), Departamento de Epidemiologia, Faculdade de Saude Publica, Universidade de Sao Paulo, Sao Paulo, SP, BR; II Departamento de Cirurgia, Faculdade de Medicina FMUSP, Universidade de Sao Paulo, Sao Paulo, SP, BR; III Hospital AC Camargo Cancer Center, Sao Paulo, SP, BR

**Keywords:** Abdominal Aortic Aneurysm, Mortality Trends, Cause of Death, Seasonality

## Abstract

**OBJECTIVES:**

Remarkable changes in the epidemiology of abdominal aortic aneurysm (AAA) have occurred in many countries during last few decades, which have also affected Brazilian mortality concurrently. This study aimed to investigate mortality trends related to AAA mortality in Brazil from 2000 to 2016.

**METHODS:**

Annual AAA mortality data was extracted from the public databases of the Mortality Information System, and processed by the Multiple Cause Tabulator.

**RESULTS:**

In Brazil, 2000 through 2016, AAA occurred in 69,513 overall deaths; in 79.6% as underlying and in 20.4% as an associated cause of death, corresponding to rates respectively of 2.45, 1.95 and 0.50 deaths per 100,000 population; 65.4% male and 34.6% female; 60.6% in the Southeast region. The mean ages at death were 71.141 years overall, and 70.385 years and 72.573 years for men and women, respectively. Ruptured AAA occurred in 64.3% of the deaths where AAA was an underlying cause, and in 18.0% of the deaths where AAA was an associated cause. The standardized rates increased during 2000-2008, followed by a decrease during 2008-2016, resulting in an average annual percent change decline of -0.2 (confidence interval [CI], -0.5 to 0.2) for the entire 2000-2016 period. As associated causes, shock (39.2%), hemorrhages (33.0%), and hypertensive diseases (26.7%) prevailed with ruptured aneurysms, while hypertensive diseases (29.4%) were associated with unruptured aneurysms. A significant seasonal variation, highest during autumn and followed by in winter, was observed in the overall ruptured and unruptured AAA deaths.

**CONCLUSIONS:**

This study highlights the need to accurately document epidemiologic trends related to AAA in Brazil. We demonstrate the burden of AAA on mortality in older individuals, and our results may assist with effective planning of mortality prevention and control in patients with AAA.

## INTRODUCTION

An abdominal aortic aneurysm (AAA) is a permanent and irreversible localized dilatation of the abdominal aorta, resulting in an aortic diameter greater than 1.5 times the expected normal. The most common site for AAA development is the region distal to the renal arteries, with an average diameter of approximately 2 cm. In clinical practice, if the diameter is ≥3 cm, the infrarenal aorta is considered aneurysmal (1,2). Moreover, the risk of rupture, which causes massive internal hemorrhage, is a significant concern, and, without prompt treatment, death can occur rapidly, with fatality rates reaching 82% (3).

In the past several decades, a dramatic change in the epidemiology and management of AAA has occurred in developed Western countries. The increasing incidence and mortality have been replaced by decreasing incidence and mortality, which has been termed “the epidemiological transition” in AAA (4). The decrease in deaths related to AAA is a result of reduced exposure to risk factors, use of cardioprotective and antihypertensive drugs, population screening, increased availability of diagnostic imaging, and innovative, minimally invasive endovascular treatment modalities. A decline in mortality was confirmed in most parts of the 19 countries of the World Health Organization, notwithstanding the heterogeneity of standardized trends, with the United States and United Kingdom recording declines, and Hungary, Romania, Austria, and Denmark recording increases (5). However, the situation may be different in other areas of the world, such as Latin America and high-income Asian-Pacific countries, where the prevalence may be increasing (6).

A trend analysis of aortic aneurysm and dissection-related mortality in the state of São Paulo, Brazil from 1985 to 2009 revealed a significant increase in age-standardized death rates in men and women for the entire period, while certain non-significant decreases occurred from 1996 and 2004 until 2009. Regarding AAA, significantly increased annual percent changes of 2.4%, 2.4%, and 3.2% were verified for total mentions in death certificates, overall, and in men and women, respectively (7). Based on the three studies referred to in the above analysis, a literature search for observational studies on prevalence and mortality rates in non-European populations from 1983 to 2013 found a prevalence trend in Brazil over a period of 16 years from 2.1% to 6% (8-11).

This study aimed to investigate the trends and causes of death related to AAA mortality in Brazil from 2000 to 2016 using a multiple-cause-of-death methodology.

## METHODS

Brazil, officially named the Federative Republic of Brazil, is the fifth largest country in the world, covering a total territory of 8.5 million km^2^, with the sixth largest population, with an estimated 210 million inhabitants as of 2019. The country is politically and administratively divided into 27 federated units (26 states and the Federal District) and 5,570 municipalities. The 27 federated units are grouped into five geographic regions: North, Northeast, Southeast, South, and Central-West.

The annual mortality data was extracted from the public multiple-cause-of-death databases of the Mortality Information System (Sistema de Informações sobre Mortalidade [SIM]) located at the Brazilian Unified Health System Information Technology Department (Departamento de Informática do Sistema Único de Saúde [DATASUS]), and the Ministry of Health (MS) (12). We selected all deaths in which AAA was listed on any line or in either part of the International Form of Medical Certificate of Cause-of-Death (the medical certification section of the death certificate), irrespective of whether it was characterized as the underlying cause of death or as an associated (non-underlying) cause. Complications of the underlying cause (Part I of the medical certification section) and contributing causes (Part II of the medical certification section) were jointly designated as associated (non-underlying) causes of death (13). We employed the 2000-2016 mid-year estimates of the population for Brazil, discriminated by year, sex, age group, and Brazilian region.

According to the International Classification of Diseases and Related Health Problems, Tenth Revision (ICD-10), the AAA as a cause of death rubrics included four character subcategories codes: I71.3, abdominal aortic aneurysm, ruptured; I71.4, abdominal aortic aneurysm, without mention of rupture; I71.5, thoracoabdominal aortic aneurysm, ruptured; I71.6, thoracoabdominal aortic aneurysm, without mention of rupture; I71.8, aortic aneurysm of unspecified site, rupture; and I71.9, aortic aneurysm of unspecified site, without mention of rupture (14).

To reconstruct the morbid process leading to death, all causes of death listed in the medical certification section of the death certificate were considered, including those classified as ill-defined, equated as such, or considered by the World Health Organization (WHO) as modes of death (13,14).

Records included in the mortality databases contain fields such as those appearing on the official Brazilian death certificate. We also created auxiliary fields for the study of multiple causes, including a field designed to contain a single &quot;string&quot; of characters composed of the codes entered on lines (a), (b), (c), and (d) of Part I and Part II of the medical certification section of the death certificate.

The causes of death were automatically processed with the Underlying Cause Selector (Seletor de Causa Básica SCB) software (15). Automatic processing involves the use of algorithms and decision tables that incorporate the WHO mortality standards and the etiological relationships among the causes of death. The expressions “death from” and “death due to” refer to the underlying cause-of-death, whereas “deaths with a mention of” and “mortality related to” refer to the listing of a given condition, either as the underlying cause or as an associated cause. The causes of death evaluated in the present study were those mentioned in the medical certification section, which are known internationally as “*entity axis codes,*” defined and presented under the structure and headings of the ICD (16).

Using mortality rates, proportions, and historical trends, we studied the distributions of the following variables: sex, age at death (in 5-year age groups), year of death, underlying cause of death, associated (non-underlying) cause(s) of death, total mentions of each cause of death, mean number of causes listed per death certificate, seasonal variation of deaths, and geographical distribution of deaths. For seasonal analysis, deaths were grouped as follows: summer, December 21^st^ through March 20^th^; autumn, March 21^st^ through June 20^th^; winter, June 21^st^ through September 20^th^; and spring, September 21^st^ through December 20^th^. Medical and demographic variables were processed using the following software: dBASE III Plus, version 1.1, dBASE IV (Ashton-Tate Corporation, Torrance, CA), and Epi Info, version 6.04d (Centers for Disease Control and Prevention, Atlanta, GA), in an emulated dbDOS™ PRO 6 environment, Excel 2016 (Microsoft Corporation, Redmond, WA). We used the Multiple Causes Tabulator (Tabulador de Causas Múltiplas for Windows) program (DATASUS, Ministério da Saúde, Faculdade de Saúde Pública, Universidade de São Paulo, Brazil) and processing codes for ICD-10 (TCMWIN, version 1.6) in our presentation of the associated causes and of the mean number of causes per death certificate (17).

To present the associated causes listed on the death certificates on which AAA was identified as the underlying cause, we prepared special lists showing the causes involved in the respective natural histories (1,2) as well as those mentioned with the greatest frequency. The duplication or multiplication of causes of death was avoided when these were presented in the abbreviated lists. The number of causes depends on the breadth of the class (subcategory, category, grouping, or chapter of the ICD-10); therefore, if two or more causes mentioned in the medical certification section were included in the same class, only one cause was computed (16,17).

The mortality rates (per 100,000 population) for AAA were calculated by year and for the study period (2000-2016) as a whole; the rates were calculated based on the number of deaths that had been identified as an underlying or associated cause as well as on the overall number of mentions. To calculate the average mortality rate, the overall number of deaths was divided by the sum of the respective annual population counts for the 17-year study period.

The *Programa para Análisis Epidemiológico de Datos* (Epidat; Epidemiological Analysis of Data Program), version 4.2 (Dirección Xeral de Innovación e Xestión da Saúde Pública, Xunta de Galicia: http://dxsp.sergas.es, and Pan American Health Organization) was used to standardize, by the direct method, the sex and age-adjusted crude and average mortality rates for the study period as a whole, to the new WHO Standard Population (18). Crude and standardized rates were calculated for 5-year age groups.

Analysis of variance was used to compare the mean numbers of causes mentioned on the death certificate, the Kruskal-Wallis H test was used to compare the mean age at death between groups, and the chi-square goodness-of-fit test was used to analyze the uniformity within the seasonal distribution of aortic aneurysms and dissection deaths. The Joinpoint Regression Program, version 4.7.0.0, was used to evaluate the changes in age-standardized rate trends (19). Assuming a Poisson distribution, joinpoint analysis chooses the best fitting point (or points) at which the rate increases or decreases significantly and calculates the annual percent change (APC) and average annual percent change (AAPC). *p*-values <0.05 were considered significant.

## RESULTS

In Brazil, from 2000 to 2016, a total of 18.683.908 deaths were recorded, of which, 69,513 overall deaths were related to AAA, 55,328 (79.6%) had AAA as the underlying cause, and 14,185 (20.4%) had AAA as an associated (non-underlying) cause of death. These deaths corresponded to the average standardized mortality rates and CIs of 2.45 (2.35-2.49), 1.95 (1.88-1.95), and 0.50 (0.44-0.53) per 100,000 population, respectively. Male and female accounted for approximately 65.4% and 34.6% of deaths, respectively, resulting in ratios of 1.89:1, 1.91:1, and 1.81:1, for overall, underlying, and associated deaths, respectively (Table 1).

Most of the deaths occurred in the Southeast region (60.6%), followed by the South (16.4%), Northeast (13.6%), Central-West (6.6%), and North (2.8%) regions, with overall average standardized mortality rates and CIs of 3.15 (3.07-3.21), 2.50 (2.40-2.54), 1.27 (1.22-1.34), 2.80 (2.68-2.88), and 1.37 (1.31-1.44) per 100,000 population, respectively. The distribution of the qualification of causes of death and sex were comparable to the overall ones (Table 1).

**Figure 1:**
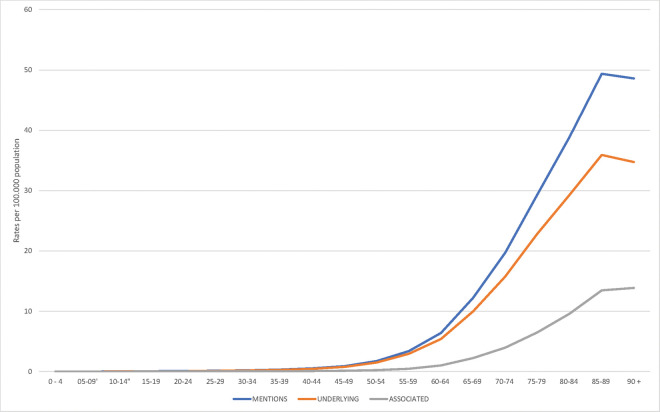
Abdominal aortic aneurysm death rates according the identified cause of death and age (Brazil, 2000-2016).

AAA deaths were observed in all age groups, although 90% occurred after 52 years of age. Death rates above 0.5 per 100.000 population began to appear in the 40 to 44 years age category, with a higher number of deaths from 75 to 79 years, but the highest rates in the 85 to 89 years age group (Figure 1). The mean age at death was strongly influenced by sex and the qualification of the cause of death, being higher in women and when AAA was considered as the associated (non-underlying) cause. For the entire period, the overall mean age at death for all mentions was 71.141 (±13.601), 70.385 (±13.098), female 72.573 (±14.397) (*p*=0.0000), underlying cause 70.517 (±13.550), associated cause 73.574 (±13.527) (*p*=0.0000), ruptured 69.408 (±14.331), without rupture 73.243 (±12.339) (*p*=0.0000), and North 68.118 (±16.361), Northeast 69.573 (±15.856), Southeast 71.595 (±13.104), South 71.844 (±12.542), and Central-West 69.752 (±13.786) (*p*=0.000) (Table 1). Ruptured AAA occurred in 54.8%, and AAA without mention of rupture in 45.2% of overall mentioned deaths. However, ruptured AAA accounted for 64.3% (males, 64.5%) as the underlying cause of death, fluctuating from 66.3% in the Southeast region to 52.0% in the North region. Among the associated causes of death, 18.0% (women, 19.4%) involved ruptured AAA, varying from 22.8% in the Northeast region to 15.2% in the South region. Of the 38,104 ruptured AAA deaths, the AAA was the underlying cause in 93.3% (male, 93.7%), and the associated cause in 6.7% (male, 6.3%) (Table 1).

**Figure 2:**
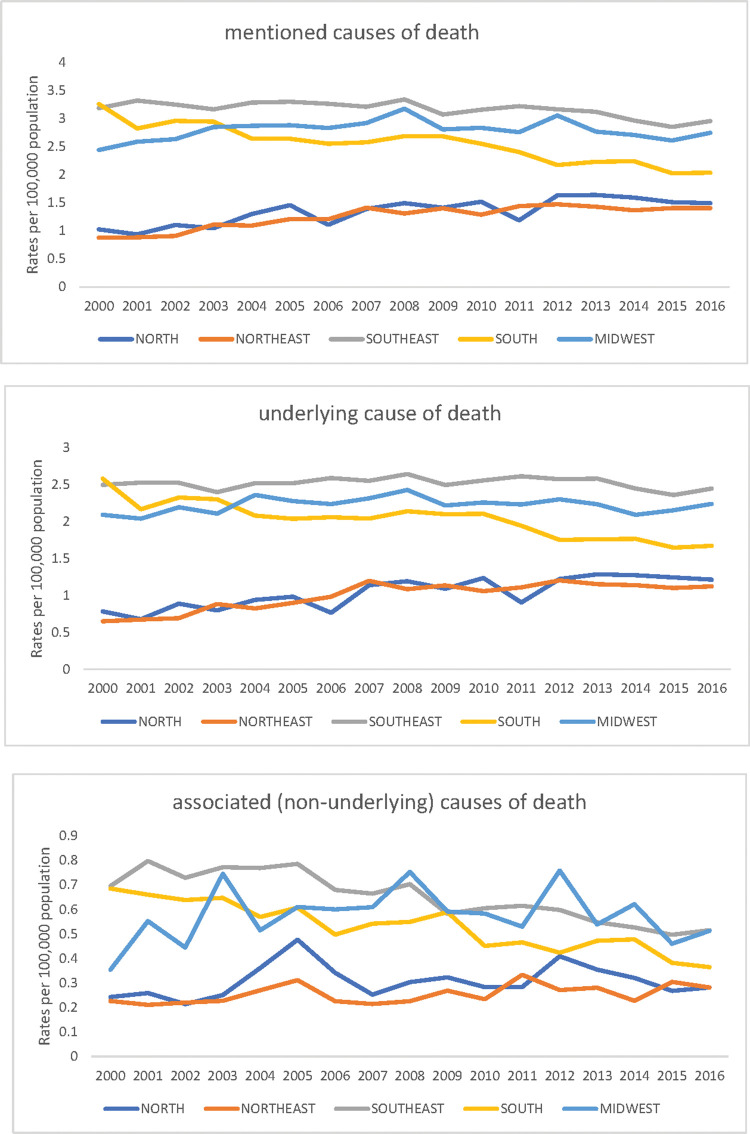
Trends of age-standardized death rates of AAA in Brazilian Regions, 2000-2016.

Joinpoint regression on overall standardized rates increased during 2000-2008, followed by a decrease in 2008-2016; this resulted in an AAPC decline of -0.2 (CI, -0.5 to 0.2) for the entire 2000-2016 study period. These figures are derived from the overall underlying and associated causes of death as well as from Brazilian regions, sex, and mention of rupture rate trends. While the age-standardized underlying cause of death rates increased and decreased as mentioned above, the associated cause rates declined continually during the entire period. The North and Northeast regions revealed increased trends; the Southeast and South regions showed decreased trends, apart from the underlying causes 2000-2012 rise in the Southeast; and the Central-West region revealed increased trends for all mentions (2000-2008) and underlying causes (2000-2007), followed by a decline, and similar rates for associated causes over the entire period. An increased trend was noticed for all mentions and underlying causes of death for women, although significance was only observed for all mentioned causes (Table 2 and Figure 2).

### Associated (non-underlying) causes in deaths related to AAA as the underlying cause

For the 17-year study, the major associated causes of deaths in which AAA was identified as the underlying cause, ruptured, and without mention of rupture, are presented in Table 3, in decreasing order of their total mentions. Shock (39.2%), hemorrhages (33.0%), hypertensive diseases (26.7%), and atherosclerosis (17.4%) prevailed with ruptured aneurysm, while hypertensive diseases (29.4%), shock (28.2%), surgical operations and procedures (23.9%), and related complications (15.0%), renal failure (13.2%), ischemic heart diseases (10.7%), and hemorrhages (10.5%) were frequently associated with aneurysms without mention of rupture.

### Underlying causes of death with AAA as an associated cause

The underlying causes of the deaths in which AAA was listed as an associated cause are presented in Table 4, according to the ICD structure and ordered after their total mentions. Major underlying causes of death were included in ICD chapters of diseases of the circulatory, respiratory, digestive systems, and neoplasms. Hypertensive and cerebrovascular diseases were most frequently reported as underlying causes of ruptured aneurysms, whereas ischemic heart diseases, diseases of the respiratory system, and neoplasms were most frequently reported as underlying causes of aortic aneurysms without mention of rupture.

### Seasonal variation

**Figure 3:**
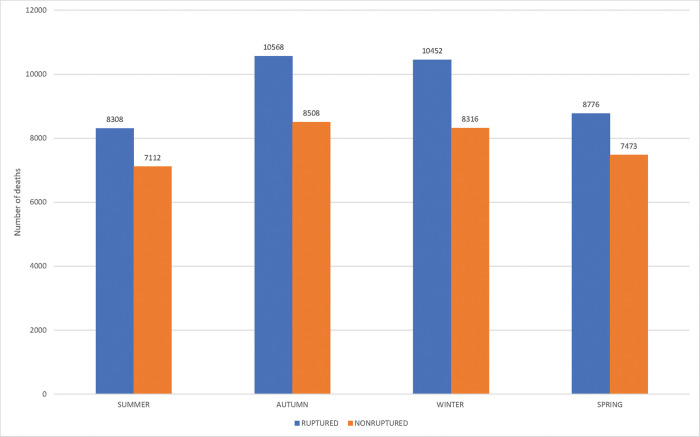
Number of deaths related to abdominal aortic aneurysms, ruptured and unruptured, according to season (Brazil, 2000-2016).

The seasonal variation in the overall ruptured AAAs, both underlying and associated causes of death, are shown in Figure 3. A significant seasonal variation was observed, with the highest frequencies during autumn (incidence rate, 27.7%), followed by winter (27.4%) (*x^2^* 135.26, *df* 3, *p*<0.00000), which was also verified in the Southeast region, with autumn (28.1%) and winter (27.5%) (*x^2^* 99.72, *df* 3, *p*<0.00000), and in the South region, with autumn (29.0%) and winter (28.2%) (*x^2^* 30.71, *df* 3, *p*<0.00000). Analogous results were observed among the unruptured AAAs. The highest significant seasonal variation was observed during autumn (27.1%), followed by winter (26.5%) for overall deaths (*x^2^* 57.18, *df* 3, *p*<0.00000). Moreover, in the Southeast region, with autumn (27.1%) and winter (26.2%) (*x^2^* 29.24, *df* 3, *p*<0.000002), while in the South region winter (27.8%), autumn (27.3%) (*x^2^* 19.92, *df* 3, *p*<0.000176) (Figure 3).

## DISCUSSION

The mortality related to AAA in Brazil was studied using the methodology of multiple causes of death; as a result, it was possible to take advantage of the resources embraced in the structure of the WHO International Form of Medical Certificate of Cause of Death, correlated guidance, and dispositions of mortality coding instructions. Almost 80% of related AAA deaths were identified as the underlying cause of death; this may reflect the correlated fatality, while nearly 65% of these deaths accounted for ruptured aneurysms, among which 93.3% were underlying causes of death. However, these values are lower than the 84.7% found for aortic aneurysm and dissection-related mortality as underlying causes in São Paulo, Brazil, from 1985 to 2009 (7). Otherwise, interesting comparisons occur between Brazilian and Washington State, in the United States. While the proportions of ruptured and without mention of rupture deaths are quite similar (54.8% and 45.2% in Brazil; 52.3% and 42.7% in Washington State), AAA is less commonly identified as the underlying cause in Washington State (64.1%) and, among ruptured aneurysms deaths, similar values when listed as underling cause of 91.3%. However, among the associated cause deaths, AAA without mention of rupture occurred only in 37.0% in Brazil, but 65.8% in Washington State. These findings suggest that AAA is a considered more suitable cause of death in Brazil than in Washington State by physicians when completing death certificates (20).

In this analysis of trends in AAA mortality in Brazil, we verified a yearly increase in all mentions during 2000-2008, followed by a decrease in 2008-2016. This initial increase in rates may be due to a greater awareness of AAA as a cause of death, the extension of mortality statistics by means of the multiple-cause-of-death methodology, and an increase in population survival, all of which have already been justified the rate increases in the São Paulo study (7). However, somehow, the rates began to influence the larger control of AAA mortality risks, mainly tobacco smoking, which remains the most important risk factor for AAA (1). The surveillance of risk and protective factors for chronic diseases by telephone disclosed the prevalence of smokers in adults (≥18 years) in Brazilian state capitals and Federal District as 9.8%, 12.3%, and 7.7%, for the overall, male, and female population in 2019, respectively, as well as an overall decline of smokers of 37.6% from 2006 to 2019. (21). The Global Burden of Disease Study 2015 mentions that Brazil was included among the 13 countries that recorded significant annualized rates of decline between 1990 and 2005 and 2005 and 2015, suggesting sustained progress in tobacco control. Among the countries with the largest number of total smokers in 2015, Brazil recorded the largest overall reduction in prevalence for both male and female daily smokers, which dropped by 56.5% (51.9-61.1) and 55.8% (48.7-61.9), respectively, between 1990 and 2015 (22).

Regarding sex, the prevalence of AAA in males has been shown to be four-to-six times higher than that of females (23). The observed ratios of male to female deaths, overall, underlying and associated, as expected, were similar to those verified in the São Paulo study for aortic aneurysms and dissection (1986 to 2009), at 1.9:1, 1.9:1, and 2.2:1, respectively.

Shock and the aggregate of hemorrhages, mentioned as the main associated causes of death related to ruptured AAA, indicate the severity of this condition. Hemorrhage is considered an important predictor of death. Due to omissions in death certification, a rupture may have even occurred in aneurysm without its mention, while hemorrhage occurred in 10.5% of the cases. Otherwise, hypertensive diseases, non-hemorrhagic shock, surgical operations, and complications of surgical and medical care were the main causes of unruptured AAA. Our finding that hypertension was the leading cause of death related to unruptured AAA is consistent with other studies that have shown its positive link as a risk factor. Indeed, hypertension was the leading cause of death of ruptured aortic aneurysms in the United States from 1996 to 2016 (24). Furthermore, a study using the Global Burden of Diseases 2017 estimates among Brazilians aged ≥25 years concluded that high systolic blood pressure was identified as the leading risk factor for cardiovascular death from 1990 to 2017 (25). The prevalence of hypertension in Brazil is remarkably high; in 2019, hypertension occurred in approximately 24.5% of the overall population, and its incidence was higher in women (27.3%) than in men (21.2%). The prevalence of hypertension by age was as follows: 31.6% in those aged 45-54 years, 45.2% in those aged 55-64 years, and 59.3% in those aged ≥65 years) (21). The current advances in smoking influence all efforts to effectively control hypertension, with the aim to prevention of AAA rupture. Mortality from AAA typically results from aneurysm rupture; however, such as has been verified with associated causes, deaths also occur after surgical and medical care, both for ruptured and unruptured aneurysms. Patients with large aneurysms commonly present with other cardiovascular risk factors, which increase their overall clinical morbidity irrespective of surgical intervention.

The issue of AAA as an associated cause of death generates doubts about the value of causes of death from death certificates. Indeed, a reviewer stresses that “It is not clear how the aneurysm can be an associated cause if it was ruptured and not the underlying cause. A ruptured aneurysm in general is highly lethal, and it is difficult to imagine that the rupture is not associated with mortality.” We agree with the reviewer that multiple-cause-of death is an overly complex matter. The problems stem from the design of the International Form of Medical Certificate of Cause of Death and the concept of underlying cause. Moreover, the value of death certificates data relies on the physician medical education and his personal subjective option, followed by coding and automatic processing of causes of death by means of decision tables that incorporate all medical world knowledge. Regarding the association of causes of death, an endless possibility of interconnecting relations may occur. An AAA may hamper the proper care and treatment of a myocardial infarction or cancer; For example, MI or cancer may be the underlying cause and AAA may be the associated cause.

A seasonal variation was observed in the country in overall deaths, and underlying and associated causes, which was more evident among ruptured that in unruptured AAA; this variation was characterized by higher mortality during autumn and winter periods, and was better defined in the Southeast and South regions, where seasonal climatic variations are more distinct. Distinct seasonal variation, with the highest frequency during winter, was observed in deaths with the underlying cause of aortic dissection, and ruptured and unruptured aortic aneurysms in the state of São Paulo from 1985 to 2009. Nevertheless, these results are in agreement with the conclusions of systematic reviews and meta-analyses, which verified that autumn and winter are significantly associated with a higher incidence of AAA rupture (26-28). Increased blood pressure levels, arterial spasm, pulmonary disease exacerbations, and passive smoking in colder weather can explain this pattern, which is also observed in other cardiovascular conditions such as cerebral and coronary ischemia.

Population mortality statistics suffer from quantity and quality problems. Furthermore, specific AAA mortality has intrinsic difficulties due to deaths without a known previous aneurysm occurring outside hospitals and without autopsy. Regarding the propriety of AAA without mention of rupture as a cause of death, some adjustment by recoding when evidence of hemorrhage was present on these death certificates to ruptured AAA was unconvincing (20). We intend to emphasize the advantages with the aim to overcome the limitations of death certificate mortality data. Estimations for 2017 show a coverage of 96.3% for the whole country, varying from 92.7% in the North region to 100% in the South region (29). Regarding quality, in a recent appraisal of the institution of multiple-cause-of-death statistics in Brazil from 2003 to 2015, the crude mean number of causes per death certificate increased from 2.81% to 3.02% (7.5%). Deaths with only one cause decreased from 20.32% to 13.75%, and ill-defined causes of death as underlying cause decreased from 12.95% to 5.67% (56.22%) (30,31). All death certificates are filled by physicians, and the verified mean number of causes mentioned on the death certificate rank among the highest in the world. Although automatic processing of mortality data is used in Brazil, the causes of death are still determined by trained nosologists who may mistakenly introduce a wrong ICD code. Finally, it is important to remember the responsibility of physicians to correctly identify the causes of death on death certificates.

## CONCLUSION

This study highlights the need to accurately document epidemiological trends related to AAA in Brazil. AAA has a significant burden on mortality in older individuals, and greater understanding of epidemiological trends will assist with the development of an effective plan for mortality prevention and control. As the first specific study on AAA mortality in Brazil, we (1) describe the progression of AAA in the Brazilian population, (2) hope to inform the medical community, (3) provide data to facilitate comparisons with Western countries, and (4) highlight important issues to further follow up AAA mortality.

## AUTHOR CONTRIBUTIONS

Santo AH participated in the design of the study, data processing, analysis and interpretation, and manuscript drafting. Puech-Leão P and Krutman M were involved in manuscript drafting and critically review for important intellectual content. All authors have read and approved the final version of the manuscript.

## Figures and Tables

**Table 1:** Deaths, crude and standardized averaged death rates, mean ages at death related to abdominal aortic aneurysms, by cause of death, sex, mention of rupture, and Brazilian regions (Brazil, 2000-2016).

	Underlying cause					Associated cause						Total			
BRAZIL	Male	Female	Ruptured	Unruptured	Subtotal	Male	Female	Ruptured	Unruptured	Subtotal	Male	Female	Ruptured	Unruptured	Total
Deaths	36,342	18,983	35,556	19,772	55,328	9,139	5,045	2,548	11,637	14,185	45,481	24,028	38,104	31,409	69,513
%	65.7	34.3	64.3	35.7	79.6	64.4	35.6	18.0	82.0	20.4	65.4	34.6	54.8	45.2	100.0
Crude	2.26	1.16	1.10	0.61	1.71	0.57	0.31	0.08	0.36	0.44	2.83	1.47	1.17	0.97	2.14
Standardized	2.95	1.17	1.25	0.70	1.95	0.76	0.31	0.09	0.41	0.50	3.72	1.48	1.34	1.11	2.45
Mean age	69.72	72.04	69.66	72.06	70.52	73.02	74.59	65.92	75.25	73.57	70.39	72.57	69.41	73.23	71.14
NORTH															
Deaths	961	567	795	733	1,528	258	178	91	345	436	1219	745	886	1,078	1,964
%	62.9	37.1	52.0	48.0	77.8	59.2	40.8	20.9	79.1	22.2	62.1	37.9	45.1	54.9	2.8
Crude	0.71	0.44	0.30	0.28	0.58	0.19	0.14	0.03	0.13	0.16	0.90	0.57	0.33	0.41	0.74
Standardized	1.39	0.76	0.54	0.53	1.07	0.38	0.24	0.05	0.25	0.31	1.77	1.01	0.59	0.78	1.37
Mean age	67.65	68.09	65.79	70.00	67.81	68.19	70.66	56.82	72.46	69.20	67.67	68.70	64.87	70.79	68.12
NORTHEAST															
Deaths	4,469	3,024	4,698	2,796	7,494	1,107	820	439	1,488	1,927	5,576	3,844	5,137	4,284	9,421
%	59.6	40.4	62.7	37.3	79.5	57.4	42.6	22.8	77.2	20.5	59.2	40.8	54.5	45.5	13.6
Crude	0.66	0.83	0.52	0.31	0.83	0.25	0.18	0.05	0.16	0.21	1.25	0.84	0.57	0.47	1.04
Standardized	1.38	0.72	0.63	0.38	1.01	0.35	0.19	0.00	0.01	0.26	1.72	0.91	0.69	0.58	1.27
Mean age	68.07	70.16	67.77	70.84	68.91	72.23	72.03	60.92	75.45	72.14	68.90	70.56	67.49	72.44	69.57
SOUTHEAST															
Deaths	22,342	11,226	22,247	11,323	33,570	5,624	2,914	1,510	7,028	8,538	27,966	14,140	23,757	18,351	42,108
%	66.6	33.4	66.3	33.7	79.7	65.9	34.1	17.7	82.3	20.3	66.4	33.6	56.4	43.6	60.6
Crude	3.31	1.61	1.62	0.83	2.45	0.83	0.42	0.11	0.51	0.62	4.14	2.03	1.73	1.34	3.07
Standardized	3.96	1.44	1.66	0.85	2.52	1.02	0.36	0.00	0.01	0.64	4.98	1.81	1.78	1.38	3.15
Mean age	70.04	72.70	70.09	72.58	70.93	73.40	75.78	67.78	75.59	74.21	70.72	73.33	69.95	73.73	71.60
SOUTH															
Deaths	6,140	2,922	5,532	3,530	9,062	1,561	780	355	1,986	2,341	7,701	3,702	5,887	5,516	11,403
%	67.8	32.2	61.0	39.0	79.5	66.7	33.3	15.2	84.8	20.5	67.5	32.5	51.6	48.4	16.4
Crude	2.65	1.24	1.18	0.75	1.94	0.67	0.33	0.08	0.42	0.50	3.32	1.57	1.26	1.18	2.44
Standardized	3.11	1.12	1.21	0.77	1.98	0.82	0.30	0.00	0.01	0.51	3.93	1.41	1.29	1.21	2.50
Mean age	70.38	73.02	70.54	72.31	71.23	73.70	75.23	68.03	75.32	74.21	71.06	73.48	70.39	73.39	71.84
MIDWEST															
Deaths	2,430	1,244	2,284	1,390	3,674	589	353	153	790	943	3,019	1,597	2,437	2,180	4,617
%	66.1	33.9	62.2	37.8	79.6	62.5	37.4	16.2	83.8	20.4	65.4	34.6	52.8	47.2	6.6
Crude	2.07	1.06	0.97	0.59	1.57	0.50	0.30	0.07	0.34	0.40	2.57	1.36	1.04	0.93	1.97
Standardized	3.11	1.43	1.37	0.85	2.22	0.78	0.41	0.01	0.02	0.58	3.88	1.83	1.45	1.34	2.80
Mean age	68.99	70.17	68.52	70.82	69.39	71.12	71.26	62.39	72.89	71.19	69.41	70.41	68.13	71.57	69.75

Source: Ministry of Health, Unified Health System Information Technology Department. Crude and standardized rates per 100,000 population. Mean age at death in years.

**Table 2:** Abdominal aortic aneurysm trends and Joinpoint Regression Analysis^a^ by cause-of-death, sex, and mention of rupture (Brazil, 2000-2016).

	Global 2000-2016			Trend 1			Trend 2		
Abdominal aortic aneurysm variables	Rates^b^	AAPC	CI	Years	APC	CI	Years	APC	CI
CAUSES OF DEATH									
Overall causes of death									
All mentions	2.45	-0.2^c^	-0.5 to 0.2	2000-2008	0.9^c^	0.4 to 1.5	2008-2016	-1.3^c^	-1.8 to -0.7
Underlying cause-of-death	1.95	0.3	-0.1 to 0.7	2000-2008	1.4^c^	0.7 to 2.2	2008-2016	-0.9^c^	-1.5 to -0.3
Associated cause-of-death	0.50	-1.9^c^	-2.5 to -1.3	2000-2016	-1.9^c^	-2.5 to -1.3			
Brazilian regions									
North: All mentions	1.37	2.6^c^	1.4 to 3.8	2000-2016	2.6^c^	1.4 to 3.8			
North: Underlying cause-of-death	1.07	3.2^c^	1.9 to 4.5	2000-2016	3.2^c^	1.9 to 4.5			
North: Associated cause-of-death	0.31	0.5	-1.8 to 2.8	2000-2016	0.5	-1.8 to 2.8			
Northeast: All mentions	1.27	3.3^c^	2.2 to 4.4	2000-2007	7.0^c^	4.7 to 9.4	2007-2016	0.5	-0.7 to 1.7
Northeast: Underlying cause-of-death	1.01	3.8^c^	2.7 to 4.9	2000-2007	8.6^c^	6.0 to 11.1	2007-2016	0.2	-1.0 to 1.4
Northeast: Associated cause-of-death	0.26	1.5^c^	0.0 to 2.9	2000-2016	1.5^c^	0.0 to 2.9			
Southeast: All mentions	3.15	-0.7^c^	-0.1 to 0.3	2000-2016	-0.7^c^	-0.1 to 0.3			
Southeast: Underlying cause-of-death	2.52	-0.3	0.3 to -0.9	2000-2012	0.3	-0.1 to 0.8	2012-2016	-0.2	-4.1 to 0.1
Southeast: Associated cause-of-death	0.64	-2.8^c^	-3.5 to 02.2	2000-2016	-2.8^c^	-3.5 to 02.2			
South: All mentions	2.50	-2.5^c^	-3.0 to -2.0	2000-2016	-2.5^c^	-3.0 to -2.0			
South: Underlying cause-of-death	1.98	-2.2^c^	-2.8 to -1.7	2000-2016	-2.2	-2.8 to -1.7			
South: Associated cause-of-death	0.51	-3.4^c^	-4.2 to -2.6	2000-2016	-3.4^c^	-4.2 to -2.6			
Center-West: All mentions	2.80	0.3	-0.6 to 1.1	2000-2008	2.1^c^	0.6 to 3.7	2008-2016	-1.5^c^	-2.7 to 0.1
Center-West: Underlying cause-of-death	2.22	0.2	-0.6 to 1.1	2000-2007	1.7	-0.0 to 3.4	2007-2016	-0.9	-1.8 to 0.1
Center-West: Associated cause-of-death	0.58	0.0	-2.0 to 2.1	2000-2016	0.0	-2.0 to 2.1			
Causes of death and sex									
All mentions of cause-of-death in men	3.72	-0.6^c^	-1.1 to -0.2	2000-2008	0.5	-0.3 to 1.2	2008-2016	-1.7	-2.4 to -1.0
All mentions of cause-of-death in women	1.48	0.9^c^	0.2 to 1.6	2000-2008	2.3^c^	1.1 to 3.5	2008-2016	-0.5	-1.4 to 0.4
Underlying cause-of-death in men	2.95	-0.2	-0.6 to 0.2	2000-2008	1.0^c^	0.3 to 1.6	2008-2016	-1.3	-1.9 to -0.8
Underlying cause-of-death in women	1.17	0.9	-0.3 to 2.2	2000-2007	5.4^c^	0.9 to 10.1	2007-2016	-0.2	-0.8 to 0.5
Associated cause-of-death in men	0.76	-1.7^c^	-3.1 to -0.3	2000-2003	4.0	-3.7 to 12.2	2003-2016	-3.0^c^	-3.7 to -2.2
Associated cause-of-death in women	0.31	-1.0^c^	-1.8 to 0.1	2000-2016	-1.0^c^	-1.8 to 0.1			
Causes of death and mention of rupture									
All mentions of cause-of-death, ruptured	1.34	-0.3	-1.6 to 1.1	2000-2016	-0.3	-1.6 to 1.1			
All mentions of cause-of-death, unruptured	1.11	0.2	-0.8 to 1.2	2000-2016	0.2	-0.8 to 1.2			
Underlying cause-of-death, ruptured	1.25	0.1	-0.3 to 0.5	2000-2016	0.1	-0.3 to 0.5			
Underlying cause-of-death, unruptured	0.70	0.4	-0.1 to 0.8	2000-2009	2.0^c^	1.4 to 2.7	2009-2016	-1.7^c^	-2.5 to -1.0
Associated cause-of-death, ruptured	0.09	-6.2^c^	-8.1 to -4.3	2000-2016	-6.2^c^	-8.1 to -4.3			
Associated cause-of-death, unruptured	0.41	-0.4	-1.4 to 0.8	2000-2005	4.1^c^	0.6 to 7.7	2005-2016	-2.3^c^	-3.2 to -1.5

AAPC: Average annual percent change, CI: Confidence interval, APC: Annual percent change.

a Joinpoint analysis of trends allowed for one joinpoint.

b Rates are per 100,000 population, age-standardized to the WHO standard population.

c The annual percent change is significantly different from 0 (*p*<0.05).

**Table 3:** Associated (non-underlying) causes of death on certificates that listed abdominal aortic aneurysm as the underlying cause of death (Brazil, 2000-2016).

	Abdominal aortic aneurysm as the underlying cause of death					
	Ruptured 35,556		Unruptured 19,772		Total 55,328	
Associated (non-underlying) cause of death (ICD-10)	n	%	n	%	n	%
Shock, not elsewhere classified (R57)	13,932	39.2	5,582	28.2	19,514	35.3
Hypertensive diseases (I10-I13)	9,507	26.7	5,808	29.4	15,315	27.7
Haemorrhages^a^	11,732	33.0	2,084	10.5	13,816	25.0
Atherosclerosis (I70)	6,189	17.4	1,619	8.2	7,808	14.1
Surgical operations and other surgical procedures (Y83-Y84)	1,918	5.4	4,717	23.9	6,635	12.0
Ischemic heart diseases (I20-I25)	2,465	6.9	2,121	10.7	4,586	8.3
Renal failure (N17-N19)	1,704	4.8	2,610	13.2	4,314	7.8
Other heart diseases^c^	2,186	6.2	1,804	9.1	3,990	7.2
Complications of surgical and medical care^b^	994	2.8	2,958	15.0	3,952	7.1
Cardiorespiratory arrest (I46.9. R09.2)	1,694	4.8	1,972	10.0	3,666	6.6
Multiple organs failure (R68.8)	1,384	3.9	1,705	8.6	3,089	5.6
Septicemias (A40-A41)	770	2.2	1,832	9.3	2,602	4.7
Cerebrovascular diseases (I60-I69)	1,547	4.4	961	4.9	2,508	4.5
Diabetes mellitus (E10-E14)	1,227	3.5	920	4.7	2,147	3.9
Chronic lower respiratory diseases (J40-J47)	1,103	3.1	954	4.8	2,057	3.7
Heart failure (I50)	748	2.1	1,176	6.0	1,924	3.5
Respiratory failure, not elsewhere classified (J96)	638	1.8	1,284	6.5	1,922	3.5
Tobacco use (smoking) (F17)	1,138	3.2	664	3.4	1,802	3.3
Pneumonias (J12-J18)	527	1.5	950	4.8	1,477	2.7
Other diseases of the arteries. Arterioles, and capillaries (I72-I78)	547	1.5	864	4.4	1,411	2.6
Vascular disorders of the intestine (K55)	337	1.0	768	3.9	1,105	2.0
Neoplasms (C00-D48)	537	1.5	420	2.1	957	1.7
Other respiratory diseases of the interstitium (J80-J84)	491	1.4	304	1.5	795	1.4
Senility (R54)	389	1.1	325	1.6	714	1.3
Other respiratory diseases^d^	248	0.7	325	1.6	573	1.0
Marfan's syndrome (Q87.4)	6	0.0	11	0.1	17	0.0

Source: Ministry of Health, Unified Health System Information Technology Department.

Rubrics and codes of the International Statistical Classification of Diseases. and Related Health Problems, Tenth Revision (1996 to 2009).

a Hemorrhages (D62, D64, I31.2. .9, J94.2, K66.1, K92.0- .2, R58).

b Complications of surgical and medical care (E89.9, G97, I97, J95, K91, N99, T80-T88).

c Other heart diseases (I00-I09, I26-I28, I30-I31, I31.3-.8, I33-I42, I44.0-I46.1, I47-I49, I51).

dOther respiratory diseases (J00-J11, J20-J39, J60-J70, J85-J94.1, J94.8-9, J98).

**Table 4:** Underlying causes of death on death certificates that listed abdominal aortic aneurysm as associated (non-underlying) cause of death (Brazil, 2000-2006).

	AAA as associated (non-underlying) cause-of-death					
	Ruptured		Unruptured		Total	
Underlying causes of death (ICD-10)	n	%	n	%	n	%
**Certain infectious and parasitic diseases (A00-B99)**	**55**	**2.2**	**372**	**3.2**	**427**	**3.0**
Septicemias (A40-A41)	27	1.1	200	1.7	227	1.6
Chagas' disease (B57)	9	0.4	38	0.3	47	0.3
**Neoplams (C00-D48)**	**97**	**3.8**	**1,174**	**10.1**	**1,271**	**9.0**
Malignant neoplasms of the esophagus and stomach (C15-C16)	12	0.5	144	1.2	156	1.1
Malignant neoplasms of the bronchus and lung (C34)	13	0.5	203	1.7	216	1.5
Malignant neoplasms of the prostate (C61)	7	0.3	110	0.9	117	0.8
**Endocrine. nutritional and metabolic diseases (E00-E90)**	**158**	**6.2**	**447**	**3.8**	**605**	**4.3**
Diabetes mellitus (E10-E14)	103	4.0	268	2.3	371	2.6
**Diseases of the nervous system (G00-G99)**	**55**	**2.2**	**372**	**3.2**	**427**	**3.0**
Alzheimer’s disease (G30)	6	0.2	61	0.5	67	0.5
**Diseases of the circulatory system (I00-I99)**	**1,401**	**55.0**	**5,697**	**49.0**	**7,098**	**50.0**
Hypertensive diseases (I10-I13)	285	11.2	989	8.5	1274	9.0
Ischemic heart diseases (I20-I25)	307	12.0	2,239	19.2	2,546	17.9
Pulmonary heart diseases and diseases of pulmonary circulation (I26-I28)	10	0.4	124	1.1	134	0.9
Cardiomyopathy (I42)	46	1.8	280	2.4	326	2.3
Other cardiac arrhythmias (I44-I49)	14	0.5	142	1.2	156	1.1
Heart failure (I50)	31	1.2	295	2.5	326	2.3
Other heart diseases (I30-I40. I51)	138	5.4	356	3.1	494	3.5
Cerebrovascular diseases (I60-I69)	463	18.2	851	7.3	1,314	9.3
Atherosclerosis (I70)	34	1.3	85	0.7	119	0.8
Other diseases of arteries. Arterioles, and capillaries (I72-I78)	61	2.4	227	2.0	288	2.0
**Diseases of the respiratory system (J00-J99)**	**206**	**8.1**	**1,827**	**15.7**	**2,033**	**14.3**
Pneumonias (J12-J18)	73	2.9	677	5.8	750	5.3
Chronic lower respiratory diseases (J40-J47)	83	3.3	837	7.2	920	6.5
**Diseases of the digestive system (K00-K93)**	**214**	**8.4**	**1,001**	**8.6**	**1,215**	**8.6**
Vascular disorders of intestine (K55)	22	0.9	192	1.6	214	1.5
Peritoneal adhesions (K660)	86	3.4	18	0.2	104	0.7
**Diseases of the genitourinary system (N00-N99)**	**63**	**2.5**	**483**	**4.2**	**546**	**3.8**
Renal failure (N17-N19)	42	1.6	255	2.2	297	2.1
Urinary tract infection, site not specified (N39.0)	9	0.4	139	1.2	148	1.0
**Congenital malformations, deformations and chromosomal abnormalities** **(Q00-Q99)**	**40**	**1.6**	**59**	**0.5**	**99**	**0.7**
Marfan's syndrome (Q87.4)	15	0.6	41	0.4	56	0.4
**External causes of morbidity and mortality (V00-Y98)**	**181**	**7.1**	**219**	**1.9**	**400**	**2.8**
Surgical operations and other surgical procedures … (Y83-Y84)	26	1.0	79	0.7	105	0.7
**Other underlying causes of death**	**106**	**4.2**	**240**	**2.1**	**346**	**2.4**
**Total**	**2,548**	**100**	**11,637**	**100**	**14,185**	**100**

Source: Ministry of Health, Unified Health System Information Technology Department.

Rubrics and codes of the International Statistical Classification of Diseases and Related Health Problems, Tenth Revision.

Surgical operations and other surgical procedures as the cause of abnormal reaction of the patient, or of later complication, without mention of misadventure at the time of the procedure (Y83-Y84).
